# *Bacillus velezensis* 802 with antagonistic activity against *Aspergillus flavus*: genome characterization, secondary metabolites and antifungal mechanism

**DOI:** 10.3389/fmicb.2026.1942172

**Published:** 2026-09-30

**Authors:** Li Zhang, Miaomiao Yang, Boyuan Zhang, Yuting Li, Lizhu Shao, Ziyu Wang, Yating Wang, Yuwen Tang, Yuansen Hu, Peng Li, Zhongke Sun, Na Liu

**Affiliations:** 1 National Engineering Research Center of Wheat and Corn Further Processing, Henan University of Technology, Zhengzhou, China; 2 School of Biological Engineering, Henan University of Technology, Zhengzhou, China; 3 Chi Tian Hua Biotechnology Co., LTD., Shangqiu, Henan, China; 4 School of International Education, Henan University of Technology, Zhengzhou, China; 5 Institute for Complexity Science, Henan University of Technology, Zhengzhou, China

**Keywords:** *Aspergillus flavus*, *Bacillus velezensis*, genome, grains storage, secondary metabolites

## Abstract

**Background:**

*Aspergillus flavus* causes severs postharvest spoilage and aflatoxins contamination of agricultural commodities, posing major threats to food safety and human health and underscoring the need for safe, effective biocontrol strategies. This study aimed to identify an antagonistic endophytic bacterium against *A. flavus* and to integrate whole-genome mining, metabolomics, and mechanistic assays to elucidate the molecular basis of its antifungal activity.

**Methods:**

An endophytic isolate was identified as *Bacillus velezensis* 802. Its crude extract was evaluated *in vitro*, and SEM, CLSM, and physiological assays were used to assess hyphal morphology, membrane integrity, mitochondrial function, energy metabolism, and apoptosis. Bioinformatic analysis of the whole-genome sequence was used to predict biosynthetic gene clusters (BGCs) and their associated pathways. UHPLC–MS/MS was used to identify metabolites in the active extract.

**Results:**

*Bacillus velezensis* 802 inhibited *A. flavus* by 70.16%. The crude extract induced hyphal wrinkling and swelling, disrupted membrane integrity, reduced mitochondrial membrane potential, suppressed key energy-metabolism enzymes, and activated the Caspase-9-mediated apoptotic pathway. Genome analysis revealed a 3,951,806 bp circular chromosome containing 13 BGCs. Metabolomics identified six secondary metabolites, among which isobutyric acid showed the strongest antifungal activity.

**Conclusion:**

By coupling whole-genome mining with metabolomic profiling and mechanistic validation, this study demonstrates that *B. velezensis* 802 suppresses *A. flavus* through multi-target interference with cell structure, energy metabolism, and apoptosis. The strain and its metabolites, especially isobutyric acid, are promising biocontrol candidates for improving grain storage safety.

## 1 Introduction

*Aspergillus flavus*, a ubiquitous saprophytic fungal pathogen, is one of the major causes of the spoilage in agricultural, including crops and stored grains (Liang et al., 2022; Wei et al., 2024a). This fungus exhibits remarkable environmental adaptability and thrives particularly well under warm and humid conditions. Upon infecting host tissues, *A. flavus* consumes host nutrients, disrupts tissue integrity, and produces toxic secondary metabolites (SMs) known as aflatoxins (AFs) (Sharma et al., 2026). Animal feed commonly contains cereal ingredients such as corn and wheat, which are highly susceptible to contamination by *A. flavus*, Consequently, AFs produced by this fungus may accumulate in feed and cause severe toxicosis or even mortality in livestock (Fouad et al., 2019). Furthermore, aflatoxin B1 (AFB1) exhibits exceptional thermal stability, rendering it largely resistant to degradation during conventional cooking and food processing procedures. The accumulation of these toxins throughout the food chain poses serious health risks, including immunosuppression, teratogenesis, and mutagenesis, particularly among vulnerable populations such as infants and older adults (Gong et al., 2024; Adjovi et al., 2025). Therefore, developing sustainable and effective strategies for controlling *A. flavus* contamination is essential to ensure food security and the safety of agricultural products and animal feed.

While chemical control remains the predominant strategy for managing *A. flavus* contamination, the long-term and repeated use of synthetic fungicides (e.g., carbendazim and imazalil) has raised significant concerns. These continuous applications have led to numerous adverse effects, ranging from the emergence of fungicide-resistant pathogen populations to environmental pollution, soil micro-ecological disruption, and a decline in beneficial microbial communities (Zhao et al., 2023). To address these challenges, biological control has gained substantial traction as a cost-effective and environmentally friendly alternative for sustainable disease management (Moh et al., 2020). Specifically, *Bacillus* species stand out as premier biocontrol candidates. Their robust environmental resilience, rapid growth kinetics, and superb biosafety-coupled with their ability to produce a wide spectrum of bioactive metabolites, extracellular enzymes, and volatile organic compounds-render them highly effective against *A. flavus* (Shenglan et al., 2023; Yao et al., 2025).

*Bacillus* species are Gram-positive, spore-forming facultative saprophytes that are ubiquitously distributed in soil, on plant surfaces, and within grain-associated environments (Su et al., 2024). Various *Bacillus* species, including *Bacillus subtilis*, *Bacillus velezensis*, *Bacillus amyloliquefaciens*, *Bacillus mojavensis*, and *Bacillus megaterium*, suppress phytopathogenic fungi through diverse biocontrol mechanisms (Camele et al., 2019; Hirozawa et al., 2023) Among these species, several have also demonstrated strong antagonistic activity against *A. flavus* (Chen Y. et al., 2019; Ling et al., 2022; Li et al., 2024). Their antifungal effects are primarily mediated by the production of bioactive metabolites, such as lipopeptides, polypeptides, phenolic compounds, hydrolytic enzymes, and other antimicrobial substances, which inhibit fungal growth, colonization, and aflatoxin production (Li et al., 2021; Salazar et al., 2023). In addition, *Bacillus* spp. can suppress *A. flavus* through competition for nutrients and ecological niches, the establishment of beneficial microbial communities, and the induction of host defense responses, including modulation of antioxidant systems. Collectively, these mechanisms enhance plant resistance to disease, improve soil microbial health, contribute to sustainable disease management, and promote plant growth (González Pereyra et al., 2018; Sun et al., 2024; Yi et al., 2025).

*Bacillus velezensis* has attracted particular attention because of its multifaceted antifungal activity against *A. flavus*. SMs produced by *B. velezensis* B2 inhibited *A. flavus* growth and aflatoxin production by modulating the expression of genes associated with fungal growth and aflatoxin biosynthesis (Wu et al., 2022). Similarly, the cell-free fermentation supernatant of *B. velezensis* 906 disrupted hyphal integrity, inhibited spore germination, and promoted AFB1 degradation (Li et al., 2024). These findings indicate that *B. velezensis* can simultaneously suppress *A. flavus* growth and development and reduce aflatoxin contamination through multiple antifungal mechanisms. Despite the substantial progress in identifying microorganisms capable of suppressing *A. flavus* growth and aflatoxin production, newly isolated strains remain an underexplored reservoir of biosynthetic diversity. Comprehensive genomic characterization, together with systematic investigation of their SMs, not only provides insights into the genetic basis of antifungal activity but also accelerates the discovery of novel and effective biocontrol agents.

In this study, *B. velezensis* 802 exhibiting strong antagonistic activity against *A. flavus* was isolated, and its biocontrol potential was evaluated by assessing the inhibitory effect of its fermentation crude extract on fungal growth. Whole-genome sequencing combined with comprehensive bioinformatics analysis was performed to identify genes potentially associated with antifungal activity. The antimicrobial SMs produced by strain 802 were characterized using UHPLC-OE480-MS/MS. Furthermore, the antifungal mechanism of these SMs were systematically investigated by examining their effects on hyphal morphology, membrane integrity, intracellular reactive oxygen species (ROS) accumulation, antioxidant enzyme activity, and cellular energy metabolism. Finally, the antifungal efficacy of the SMs was further validated in coarsely ground grain substrates and animal feed to evaluate their practical potential for controlling *A. flavus* contamination.

## 2 Materials and methods

### 2.1 Microbial strains, chemicals, and culture medium preparation

*Bacillus velezensis* 802 and *A. flavus* NRRL 3357, used in this study were maintained in the laboratory of the College of Biological Engineering, Henan University of Technology, Zhengzhou, China.

Isobutyric acid, lauric acid, *p*-nitroacetophenone, methyl diethylphosphonoacetate (MDEP), estradiol valerate, and ethyl caprate were purchased from Shanghai Aladdin Biochemical Technology Co., Ltd. (Shanghai, China). The JC-1 dye for mitochondrial membrane potential analysis was purchased from Beyotime Biotechnology (Shanghai, China). The Propidium iodide (PI) staining kit, superoxide dismutase (SOD) activity assay kit, NAD-malate dehydrogenase (NAD-MDH) activity assay kit and Caspase-9 activity assay kit were purchased from Beijing Solarbio Technology Co., Ltd. (Beijing, China).

Potato dextrose agar (PDA) medium (20% potato, 2% dextrose, 2% agar, pH 7.0), Potato dextrose broth (PDB) medium (20% potato, 2% dextrose, pH 7.0), were prepared and sterilized at 115 °C for 30 min before use.

### 2.2 Screening of antagonistic strains

Strains exhibiting antagonistic activity against *A. flavus* were screened by using the plate confrontation assay. *A. flavus* was pre-cultured on PDA at 30 °C for 5 d. A single colony of *A. flavus* and each candidate antagonistic strains was separately inoculated into PDB and incubated at 30 °C with shaking at 240 rpm for 12 h. A 3 μL aliquot of the *A. flavus* seed culture was inoculated at the center of a 90 mm PDA plate, and 3 μL aliquots of bacterial cultures were inoculated at equal distances on opposite sides of the fungal inoculum. Plates inoculated with *A. flavus* alone, with an equal volume of sterile water applied to the positions corresponding to the bacterial inocula, served as the control. All plates were cultured at 30 °C until the fungal colony in the control group completely covered the entire plate.

The inhibition rate was calculated using the following equation:

$$\text{Inhibition rate}\;(\%)=(\frac{C-T}{C})\times 100\%$$

where *C* represents the control radius of the control group and *T* represents the colony radius of the treated group (Gu et al., 2022). All experiments were conducted in triplicate.

### 2.3 Preparation of crude extract and determination of its antifungal activity

The strain 802 was inoculated in 250 mL of PDB medium and incubated at 30 °C with shaking at 240 rpm for 48 h. The fermentation broth was centrifuged at 8000 rpm for 10 min to remove cells, the resulting culture supernatant was passed through a 0.45 μm membrane filter to remove residual cells. The pH the filtrate was adjusted to 2.0 using 1 M HCl, followed by overnight incubated at 4 °C to precipitate the crude extract. The precipitate was collected by centrifugation at 9000 rpm for 10 min at 4 °C and resuspended in 60% methanol to a final volume equivalent at 1/100 of the original culture volume. The suspension was neutralized to pH 7.0 using 1 M NaOH and centrifuged again at 12000 rpm for 10 min. The detailed extraction procedure was performed as previously described by Gu et al. (2022). Finally, the supernatant was filtered through a 0.22 μm nylon membrane to obtain the crude extract.

The Oxford cup assay of Zhang et al. (2022) was slightly modified to evaluate the antifungal activity of the crude extract of strain 802 against plant pathogenic fungi. Two equidistant wells were punched on opposite sides of the center of a double-layer PDA plate, and 150 μL of crude extract was added to each well. An equal volume (150 μL) of deionized water or 60% methanol were used as the negative control. The remaining experimental procedures were identical to those described for the dual-culture assay. All experiments were performed in triplicate.

### 2.4 Evaluation of the inhibitory effect of the crude extract of strain 802 on *Aspergillus flavus* colony formation in coarsely ground grain substrates and feed

Wheat and maize grains were surface-disinfected with 1% NaCIO (*v/v*), rinsed three times with sterile distilled water, aseptically dried at 60 °C for 6 h, and their moisture content was adjusted to 20%. The grains were ground for 20 s, sieved through a 40-mesh screen, and the retained fractions was used as coarse wheat and maize powder. Fifteen grams of wheat, corn and feed powder were separately weighed and inoculated with an *A. flavus* spore suspension to obtain a final concentration of 1 × 10^5^ spores/g. The moisture content of each sample was then adjusted to 20%. The treatment group received 2% (*v/w*) crude extract of strain 802, whereas the control group received no crude extract. All samples were incubated under the identical conditions for 3 d. Following robust mycelial growth, serial dilutions were prepared up to 10^−4^ and spread-plated onto PDA. Colony-forming units (CFUs) were enumerated after 2 d of incubation. All experiments were performed in triplicate.

### 2.5 Microscopic characterization of the inhibitory effect of *Bacillus velezensis* 802 on the growth of *Aspergillus flavus* mycelia

*Aspergillus flavus* was inoculated into 100 mL of PDB medium and cultured at 30 °C for 24 h with shaking at 240 rpm. Subsequently, 10 mg of wet mycelia were harvested, resuspended in 980 μL of PDB, and treated with 20 μL of crude extract (prepared as described in Section 2.3; 1:50, *v/v*). The suspension was then cultured at 30 °C for 4 h. The control group received an equal volume of PDB medium.

After treatment, the mycelia were collected by centrifugation and washed three times with 0.1 M PBS. The samples were fixed overnight at 4 °C in 2.5% (*v/v*) glutaraldehyde and subsequently washed three times with PBS for 15 min each, followed by two washes with ultrapure water. The fixed mycelia were sequentially dehydrated with 30, 50, 70, and 90% ethanol for 10–15 min at each concentration, followed by two dehydration steps with 100% ethanol for 15 min each. The ethanol was then gradually replaced with tert-butanol using ethanol/tert-butanol mixtures at volume ratios of 2:1 and 1:1, followed by 100% tert-butanol, with each replacement step lasting 10 min. The mycelia were finally resuspended in an appropriate volume of tert-butanol, transferred onto aluminum foil, pre-frozen at –80 °C, and freeze-dried. The dried samples were subsequently prepared for scanning electron microscopy (SEM) observation.

### 2.6 Effects of *Bacillus velezensis* 802 crude extract on membrane integrity and mitochondrial function in *Aspergillus flavus*

*Aspergillus flavus* mycelia treated as described in Section 2.5 were subjected to PI staining to asses plasma membrane integrity following exposure to the crude extract of strain 802. The mycelia were collected, washed twice with PBS, resuspended in PBS, and incubated with PI at a final concentration of 10 μg/mL for 10 min in the dark. After staining, the mycelia were washed twice with PBS to remove unbound PI and immediately examined using fluorescence microscopy.

For mitochondrial membrane potential analysis, the mycelia were incubated with 0.5 mL of JC-1 staining solution at 37 °C for 20 min according to the manufacturer’s instructions. After incubation, the mycelia were washed twice with JC-1 staining buffer and collected by centrifugation. The collected mycelia were resuspended in 1 mL of JC-1 staining buffer, and mycelial film was prepared for observation under a confocal laser scanning microscope (CLSM).

### 2.7 Biochemical effects of *Bacillus velezensis* 802 crude extract on *Aspergillus flavus*

The activities of SOD, NAD-MDH, and Caspase-9 in *A. flavus* were determined using the corresponding commercial assay kits. Mycelia from the crude extract-treated and the control groups were prepared as described in Section 2.5. For each sample, 0.1 g of mycelia was collected and ground into a fine powder under liquid nitrogen. The activities of SOD, NAD-MDH and Caspase-9 were subsequently determined according to the manufacturers’ instructions.

### 2.8 Genome sequencing, assembly, functional annotation, and phylogenomic identification

Whole genome sequencing of strain 802 was performed by Beijing Qingke Biotechnology Co., Ltd. (Beijing, China) using a hybrid sequencing strategy combining Illumina NovaSeq short-read sequencing and Oxford Nanopore Technologies (ONT) long-read sequencing (Deamer et al., 2016). For ONT sequencing, high-quality genomic DNA was used for library construction with the SQK-LSK109 ligation sequencing kit according to the manufacturer’s protocol. Raw Nanopore signals were base-called using Guppy v3.2.6, and low-quality and short reads (< 2000 bp) were removed prior to genome assembly. The filtered ONT long reads were *de novo* assembled using Canu v1.5 (Guifang and Liu, 2022). The initial assembly was polished with ONT long reads using Racon v3.4.3, followed by genome circularization and adjustment of the chromosome start position using Circlator v1.5.5 (Martin et al., 2015; Robert et al., 2017). The resulting assembly was further polished using Illumina short reads with Pilon v1.22. Assembly completeness and contamination were assessed using BUSCO v5.8.2 and CheckM v1.1.3, respectively (Donovan et al., 2015; Walker et al., 2017; Seppey et al., 2019).

Structural and functional annotation of the assembled genome was performed using multiple bioinformatic tools and databases. Coding sequence (CDS) was predicted using Prodigal v2.6.3. tRNA genes were identified using tRNAscan-SE v2.0 (Patricia et al., 2021), whereas rRNA genes were predicted using Infernal v1.1.3 based on covariance models from the Rfam database (Deepank et al., 2020). Repetitive sequences were identified using RepeatMasker v4.0.5 (Benson, 1999).

Functional annotation of the predicted protein-coding genes was performed using sequence similarity searches against multiple databases, including Gene Ontology (GO), Kyoto Encyclopedia of Genes and Genomes (KEGG), eggNOG, NCBI non-redundant protein (NR), UniProt, Swiss-Prot, and Pfam. Putative biosynthetic gene clusters (BGCs) associated with secondary metabolite biosynthesis were identified and annotated using antiSMASH v7.1.0.

To determine the phylogenomic position and taxonomic identity of strain 802, its genome and those of representative *Bacillus* type strains, including *B. velezensis* FZB42ᵀ, *Bacillus siamensis* KCTC 13613, *B. amyloliquefaciens* NRRL B-14393ᵀ, *Bacillus nakamurai* NRRL B-41091ᵀ, and *Bacillus atrophaeus* NBRC 15539ᵀ, were subjected to genome-based phylogenetic analysis using the Type (Strain) Genome Server (TYGS)^1^.

### 2.9 Identification of substances in crude extract of strain 802

The chemical constituents of the crude extract obtained from strain 802 were separated and characterized using by ultra-high performance liquid chromatography coupled with Orbitrap Exploris 480 tandem mass spectrometry (UHPLC-OE480-MS/MS). Chromatographic separation was performed using an Acclaim™ RSLC 120 C18 column (2.1 × 150 mm, 2.2 μm 120 Å), with water as mobile A and acetonitrile as mobile phase B. The following linear gradient program was applied: 0.0–10.0 min, 60–20% A, 40–80% B; 10.0–15.0 min, 20–5% A, 80–95% B. The column temperature was maintained at 40 °C, the injection volume was 4 μL, and the flow rate was set at 0.3 mL/min. Mass spectrometric detection was performed in negative electrospray ionization mode (ESI^−^) over an *m/z* range of 100–2000.

### 2.10 Activity of *Bacillus velezensis* 802 SMs against *Aspergillus flavus*

Among the six selected SMs, isobutyric acid, ethyl decanoate were liquids and were tested in their undiluted forms, whereas lauric acid, MDEP, estradiol valerate and *p*-nitroacetophenone were solids and were dissolved in 60% methanol to prepare working solutions at a concentration of 0.2 mg/mL. An aliquot (30 μL) of each test compound or solution was added to the corresponding Oxford cup. For the solid compounds prepared in 60% methanol, an equal volume (30 μL) of 60% methanol was used as the solvent control. The plates were subsequently incubated and evaluated under the conditions described in Section 2.2.

### 2.11 Data analysis

Data are presented as mean ± standard of three independent experiments. Raw data were organized and initially processed using Excel 2021. Statistical analyses were performed using GraphPad Prism 10. Differences among treatment groups were analyzed by one-way analysis of variance (ANOVA), followed by Duncan’s multiple range test, with *p* < 0.05 considered statistically significant. Different lowercase letters indicate significant differences among treatments.

## 3 Results

### 3.1 Screening of bacterial isolates with antifungal activity against *Aspergillus flavus*

A total of 30 bacterial isolates were obtained from wheat seed samples via the dilution plating method, among which 6 strains were verified to exert antifungal activity against *A. flavus* through plate confrontation assays (Figure 1). The inhibition rates of strains 802, 266, 8, 203, 2,682 and 213 on *A. flavus* were 70.16, 66.79, 64.66, 64.26 62.66 and 61.85%. The results showed that strain 802 had the best inhibitory effect on *A. flavus*. Based on its comparatively strong antifungal activity, strain 802 was selected for subsequent identification and further characterization.

**Figure 1:**
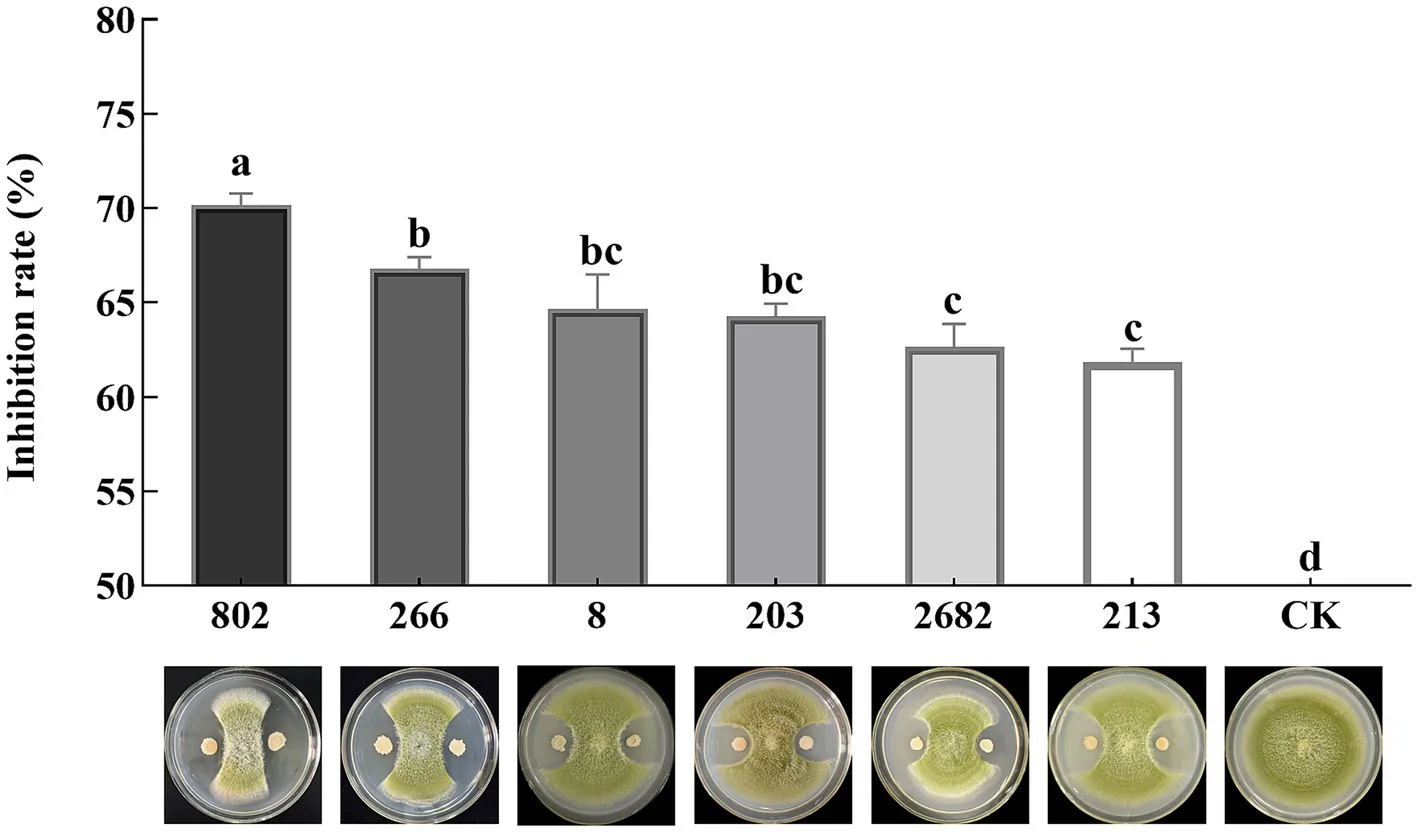
Inhibitory effect of 6 strains of bacteria on *Aspergillus flavus*. Different lowercase letters indicate significant differences in the comparison of the same indicator (*p* < 0.05).

### 3.2 Inhibitory effects of the crude extract from strain 802 on mycelial growth and conidiation of *Aspergillus flavus*

The inhibitory effects of fermentation crude extract from strain 802 and the 60% methanol against the mycelial growth of *A. flavus* were evaluated (Figure 2A). The results showed that 60% methanol had no significant inhibitory effect on *A. flavus*. Following treatment with the crude extract, the inhibition rates were 9.35, 11.47, 15.00, 29.60, and 47.52% on 2, 4, 6, 8, and 10 d, respectively (Figure 2B). The inhibition rate reached the highest on the 10th d.

**Figure 2:**
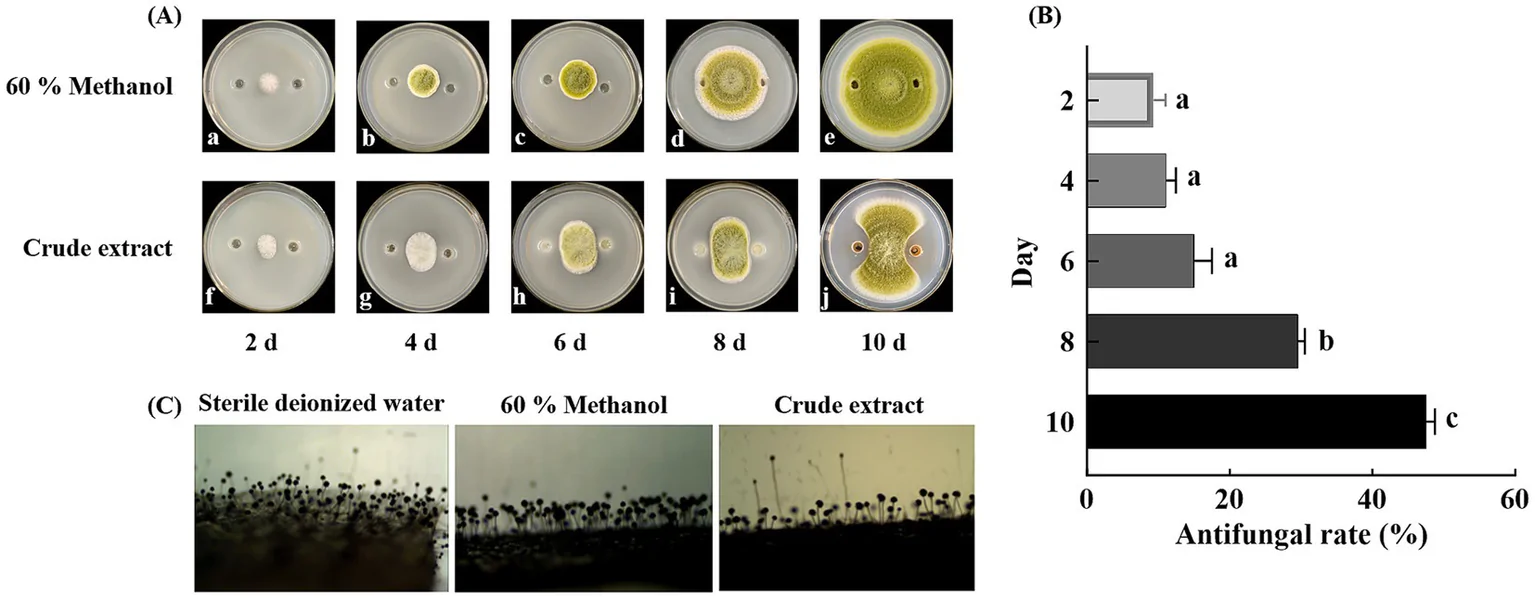
Antifungal effects of the crude extract from strain 802 against *Aspergillus flavus*. **(A)** Colony growth of *Aspergillus flavus* in the presence of 60% methanol **(a-e)** or the crude extract from strain 802 (f-j) at the corresponding observation time points. Images from left to right represent days 2, 4, 6, 8, and 10 after treatment. **(B)** Mycelial growth inhibition rates of *Aspergillus flavus* following treatment with the crude extract from strain 802 on days 2, 4, 6, 8, and 10. **(C)** Effects of the crude extract from strain 802 on the morphological development and conidiation of *Aspergillus flavus*. Different lowercase letters indicate significant differences among treatments for the same parameter (*p* < 0.05).

To further investigate the effects of the crude extract on the morphological development and conidiation of *A. flavus*, samples from each treatment group were sectioned and examined under a light microscope using a 40 × objective lens, with 60% methanol serving as the solvent and blank controls. Comparable conidiophore morphology and conidial abundance were observed in the 60% methanol groups (Figure 2C). In contrast, treatment with the crude extract markedly reduced conidial abundance and induced conspicuous morphological alterations in the conidiophores, including abnormal elongation of some conidiophores. These morphological changes suggest that the crude extract from strain 802 interfered with normal conidiophore development and conidiation in *A. flavus*.

### 3.3 Antifungal activity of strain 802 crude extract against *Aspergillus flavus* in feed, and coarsely ground grain substrate

The antifungal efficacy of the crude extract of strain 802 against *A. flavus* in coarsely ground corn, coarsely wheat, and compound feed was evaluated by visual assessment of fungal growth and quantitative colony enumeration. Extensive visible mold growth was observed in all untreated control substrates, whereas substrates treated with the crude extract exhibited markedly less fungal growth, particularly in coarsely ground corn, in which almost no visible mold colonies were observed (Figure 3A). Quantitative colony enumeration further demonstrated that treatment with the crude extract significantly reduced the recoverable population of *A. flavus* in all three substrates, with reductions of 91.82, 45.85, and 28.51% in coarsely ground corn, coarsely ground wheat, and compound feed, respectively, relative to the corresponding untreated controls (Figure 3B). These findings demonstrate that the crude extract of strain 802 retained antifungal activity across different grain- and feed-based matrices, although its efficacy varied considerably among substrates. The pronounced inhibitory effect observed in coarsely ground corn suggests that the crude extract may have potential as a naturally derived antifungal preparation for controlling *A. flavus* contamination in susceptible agricultural commodities.

**Figure 3:**
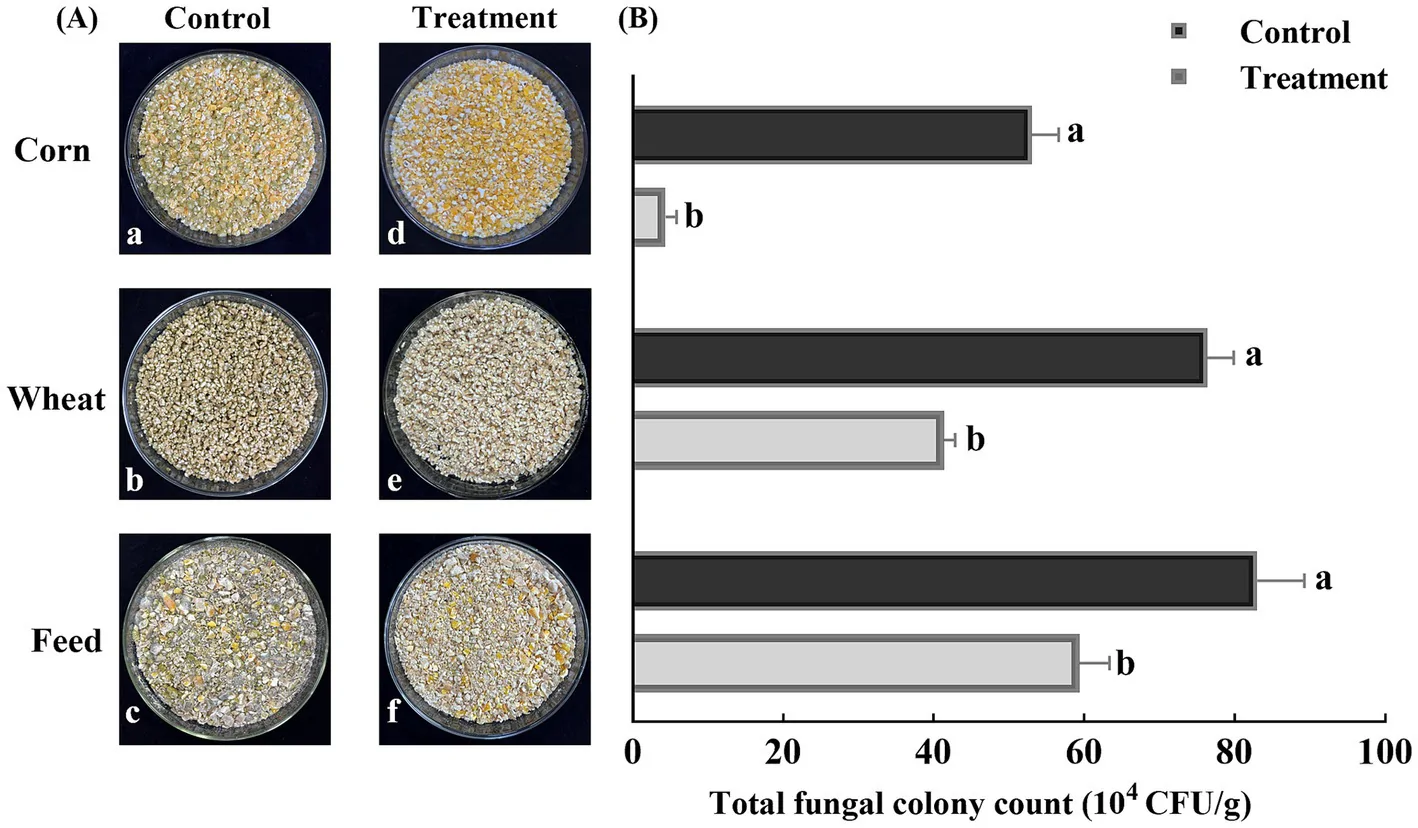
Antifungal activity of the crude extract from strain 802 against *Aspergillus flavus* in coarsely ground corn, coarsely ground wheat, and compound feed. **(A)** Representative appearance of the three substrates inoculated with *Aspergillus flavus* spores in the absence or presence of the crude extract. Panels a-c represent the untreated controls of coarsely ground corn, coarsely ground wheat, and compound feed, respectively, whereas panels d-f represent the corresponding crude-extract-treated groups. **(B)** Quantitative enumeration of recoverable *Aspergillus flavus* colonies in the three substrates. Different lowercase letters indicate significant differences among treatments for the same parameter (*p* < 0.05).

### 3.4 Genomic characterization of *Bacillus velezensis* 802

#### 3.4.1 Genome analysis and phylogenetic identification of *Bacillus velezensis* 802

To characterize the genomic features of strain 802, whole-genome sequencing and genome annotation were performed. The assembled genome of strain 802 was 3,951,806 bp in length with a GC content of 46.61% and contained 3,812 predicted CDSs and 86 tRNA genes (Figure 4A). The combined length of the predicted CDSs was 3,503,907 bp, accounting for 88.67% of the entire genome.

**Figure 4:**
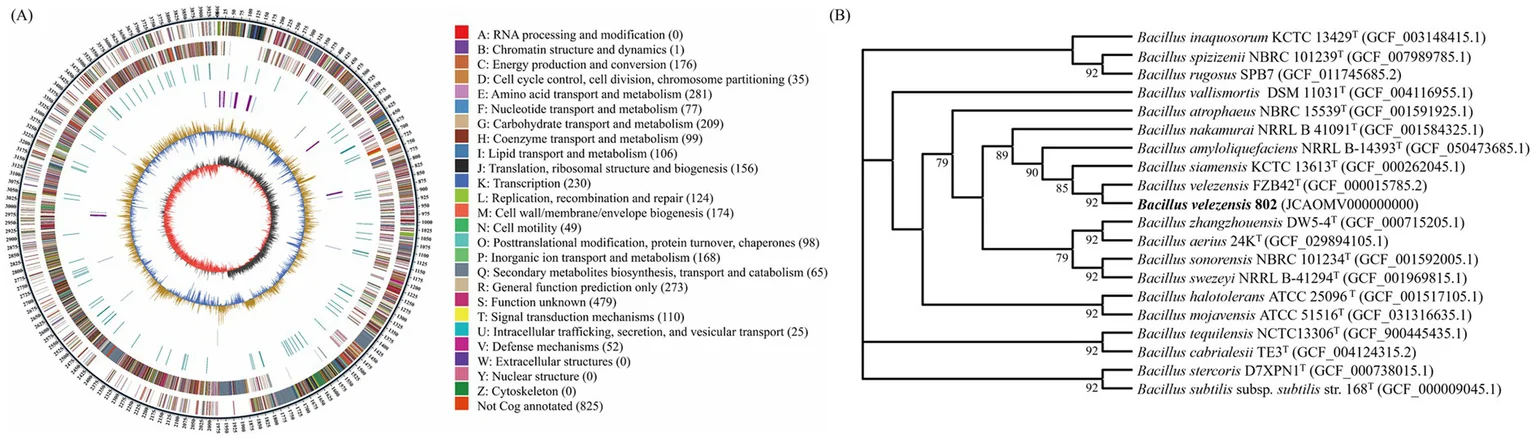
Genome organization and phylogenetic analysis of *Bacillus velezensis* 802. **(A)** Circular genome map of strain 802. From the outermost to the innermost ring, the circles represent genome size, genes encoded on the forward and reverse strands and colored according to their COG functional categories, repetitive sequences, tRNA and rRNA genes, GC content, and GC skew, respectively. **(B)** Whole-genome-based phylogenetic tree showing the evolutionary relationship of strain 802 with representative *Bacillus* strains.

To determine the taxonomic position of strain 802, a phylogenetic analysis was conducted using whole-genome sequences of strain 802 and representative related *Bacillus* strains. The whole-genome-based phylogenetic tree showed that strain 802 clustered within the same clade as *B. velezensis* FZB42ᵀ, supporting its identification as *B. velezensis* (Figure 4B).

To contextualize the genomic traits of strain 802, its features were compared against representative *Bacillus* strains sharing high 16S rRNA gene sequence similarity, including *B. velezensis* FZB42ᵀ, *B. siamensis* KCTC 13613ᵀ, *B. amyloliquefaciens* NRRL B-14393^T^, *B. nakamurai* NRRL B-41091ᵀ, and *B. atrophaeus* NBRC 15539ᵀ (Table 1). Strain 802 contained 3,812 CDSs, which was comparable to the number in *B. velezensis* FZB42ᵀ (3,762) but lower than that in *B. amyloliquefaciens* NRRL B-14393^T^ (4,312). The GC content of strain 802 (46.61%) was comparable to those of *B. velezensis* FZB42ᵀ (46.5%) and *B. siamensis* KCTC 13613ᵀ (46.5%) but higher than those of the other strains included in the comparison. Strain 802 contained 86 tRNA genes, the same number as that in *B. siamensis* KCTC 13613ᵀ. The contig N50 value of strain 802 was 3,951,806 bp, comparable to that of *B. velezensis* FZB42ᵀ (3,918,596 bp) and higher than those of the other strains included in the comparison.

**Table 1:** Comparative genomic features of *Bacillus velezensis* 802 and representative *Bacillus* strains.

Taxon name	Strain name	Contigs	Genome size (bp)	GC content (%)	CDSs	tRNA genes	Contig N50 (bp)
*B. velezensis*	802	1	3,951,806	46.6	3,812	86	3,951,806
*B. velezensis*	FZB42^T^	1	3,918,596	46.5	3,762	88	3,918,596
*B. siamensis*	KCTC 13613^T^	1	3,779,696	46.5	3,695	86	589,476
*B. amyloliquefaciens*	NRRL B-14393^T^	1	4,205,553	46.0	4,312	85	1,977,446
*B. nakamurai*	NRRL B-41091^T^	1	3,689,277	45.5	3,678	82	142,440
*B. atrophaeus*	NBRC 15539^T^	1	4,158,197	43.0	4,193	63	300,882

#### 3.4.2 eggNOG annotation of strain 802

eggNOG annotation was performed to functionally classify the predicted protein-coding genes of strain 802 based on orthologous relationships. A total of 2,987 CDSs were assigned to 21 eggNOG functional categories, accounting for 78.52% of the predicted protein-coding genes in the genome (Figure 5). Among these categories, function unknown (S) contained the largest number of genes. Excluding this category, the most highly represented functional groups were amino acid transport and metabolism (E), general function prediction only (R), and transcription (K), comprising 281,273, and 230 genes, respectively. Other abundant categories included carbohydrate transport and metabolism (G), cell wall/membrane/envelope biogenesis (M), and energy production and conversion (C), containing 218,179 and 176 genes, respectively. The relatively high representation of genes associated with amino acid and carbohydrate metabolism, energy production, and transcription suggests that strain 802 possesses a diverse genetic repertoire potentially supporting nutrient utilization, energy metabolism, and transcriptional regulation. Notably, the large proportion of genes assigned to the function unknown category, together with the predicted protein-coding genes that could not be assigned to eggNOG functional categories, indicates that the genome of strain 802 contains a substantial number of genes whose biological functions remain to be characterized.

**Figure 5:**
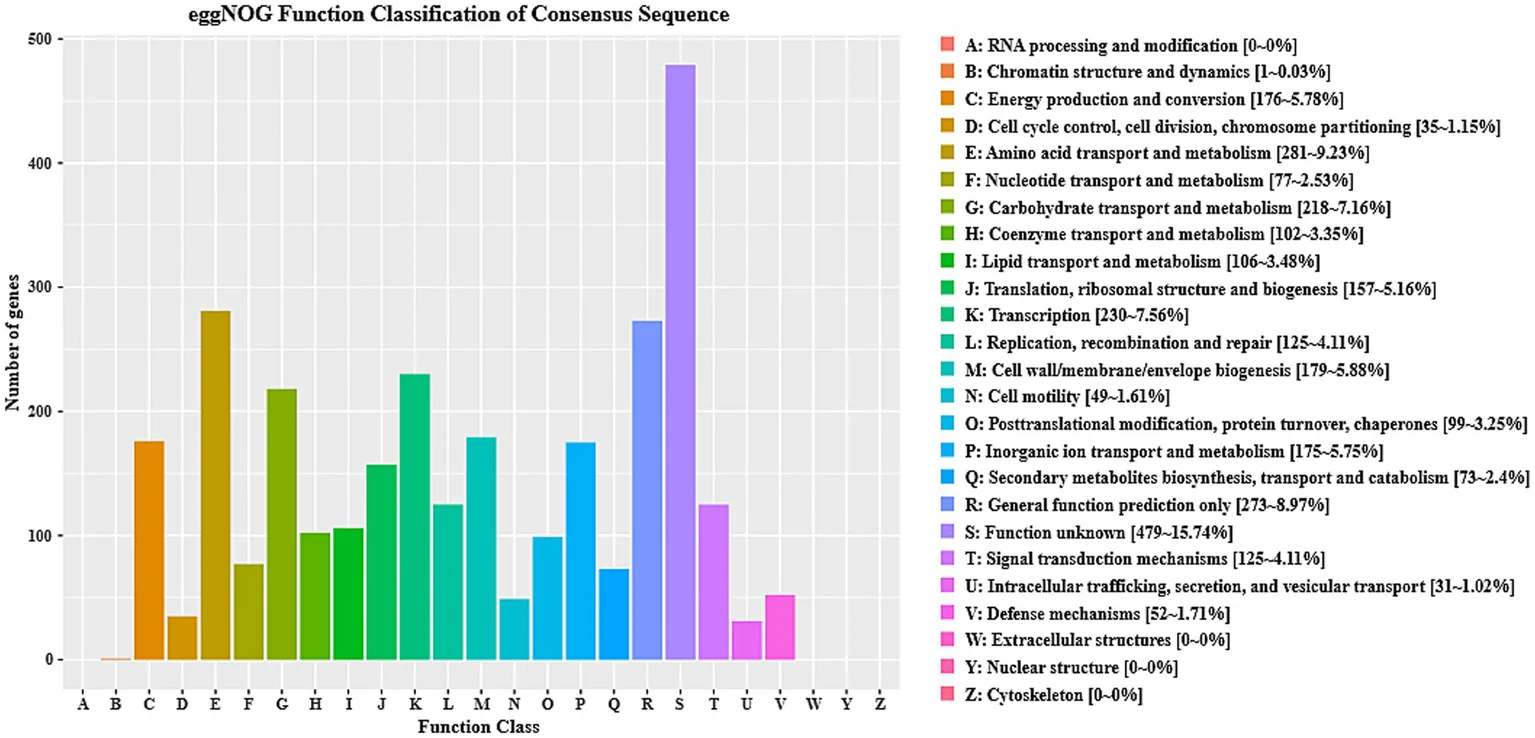
Number of strain-specific genes of 802 with general eggNOG functional categories.

#### 3.4.3 GO annotation of strain 802

GO annotation divided the identified proteins into three categories according to gene function, namely cellular component, molecular function and biological process. As shown in Table 2, a total of 2,545 coding genes were annotated in the genome of 802 strain, accounting for 66.90%. In the cell components, mainly including membrane (833 genes), membrane components (787 genes), cells (760 genes), cell components (746 genes); on the molecular function, mainly including catalytic activity (1,444 genes), binding (1,049 genes), transport activity (225 genes) and nucleic acid binding transcription factor activity (120 genes); In biological processes, including metabolic processes (1,254 genes), cellular processes (1,071 genes), single biological processes (965 genes) and localization (363 genes). These results showed that the strain had active metabolic function and cell physiological function. Strain 802 contained 79 genes involved in proteolysis (GO:0006508), 1 gene involved in siderophore biosynthetic process (GO:0019290), 72 genes involved in hydrolase activity (GO:0016787), and 1 gene had cellulase activity (GO:0008810). One gene was involved in pectin lyase (GO:0047490). Collectively, these genomic features suggest that strain 802 has the potential for protease production, siderophore biosynthesis, and polysaccharide degradation.

**Table 2:** Partial GO description of protein-coding genes in the *Bacillus velezensis* strain 802 genome.

Category	Partial GO description	Number
Cellular component	Membrane	833
	Membrane part	787
	Cell	760
	Cell part	746
Molecular function	Catalytic activity	1,444
	Binding	1,049
	Transporter activity	225
	Nucleic acid binding transcription factor activity	120
Biological process	Metabolic process	1,254
	Cellular process	1,071
	Single-organism process	965
	Localization	363

#### 3.4.4 KEGG annotation of *Bacillus velezensis* 802

KEGG functional annotation showed that the annotated genes were assigned to 40 pathways distributed among four major functional categories: Metabolism, Cellular Processes, Environmental Information Processing, and Genetic Information Processing (Figure 6). A total of 2,724 predicted protein-coding genes were assigned KEGG annotations, accounting for 71.61% of all predicted protein-coding genes in the genome. Among the annotated pathways, the two-component system, biosynthesis of amino acids, ABC transporters, carbon metabolism, and purine metabolism contained the largest numbers of annotated genes, with 143, 126, 107, 100, and 57 genes assigned to these pathways, respectively.

**Figure 6:**
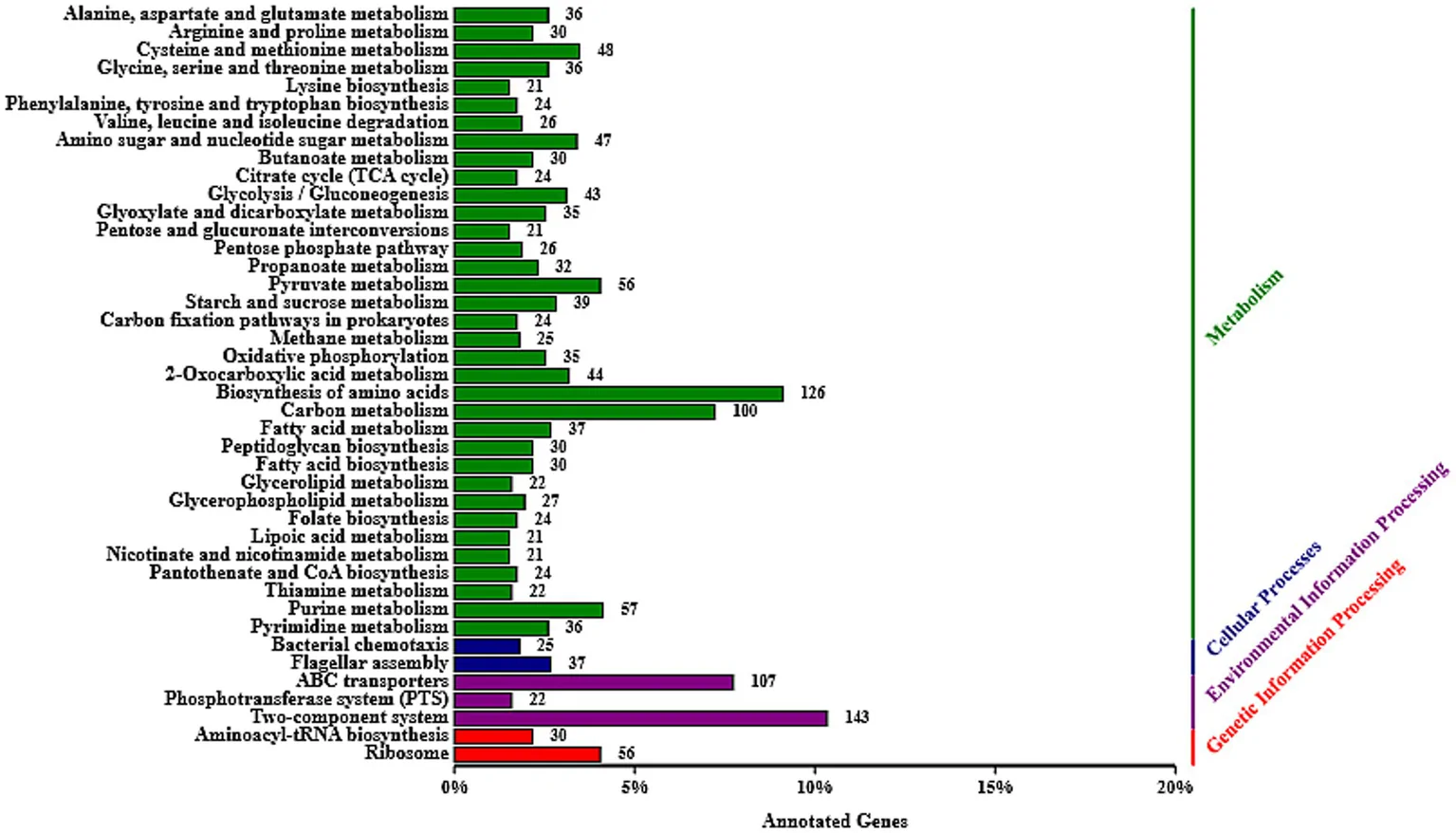
KEGG pathway classification of the annotated genes in *Bacillus velezensis* 802.

### 3.5 Effects of the crude extract from strain 802 on membrane integrity, mitochondrial membrane potential, cellular morphology, and spore germination of *Aspergillus flavus*

PI is membrane-impermeable and is therefore generally excluded from cells with intact plasma membranes; however, when membrane integrity is compromised, PI enters the cells, binds to nucleic acids, and produces red fluorescence. As shown in Figure 7A, only weak background fluorescence was observed in the control group, consistent with the maintenance of plasma membrane integrity. In contrast, intense red fluorescence was observed in *A. flavus* hyphae exposed to the crude extract from strain 802, indicating a marked increase in membrane permeability and substantial loss of plasma membrane integrity. JC-1 is a widely used fluorescent probe for assessing changes in mitochondrial membrane potential (ΔΨm). At relatively high ΔΨm, JC-1 forms aggregates within mitochondria and emits red fluorescence. Conversely, mitochondrial depolarization favors the monomeric form of JC-1, resulting in green fluorescence. Fluorescence microscopy revealed predominantly red fluorescence in untreated control hyphae, with only limited green fluorescence. In contrast, hyphae treated with the crude extract from strain 802 exhibited predominantly green fluorescence accompanied by weaker red fluorescence (Figure 7B). This shift in the JC-1 fluorescence pattern suggests that exposure to the crude extract from strain 802 induced mitochondrial membrane depolarization in *A. flavus* hyphae.

**Figure 7:**
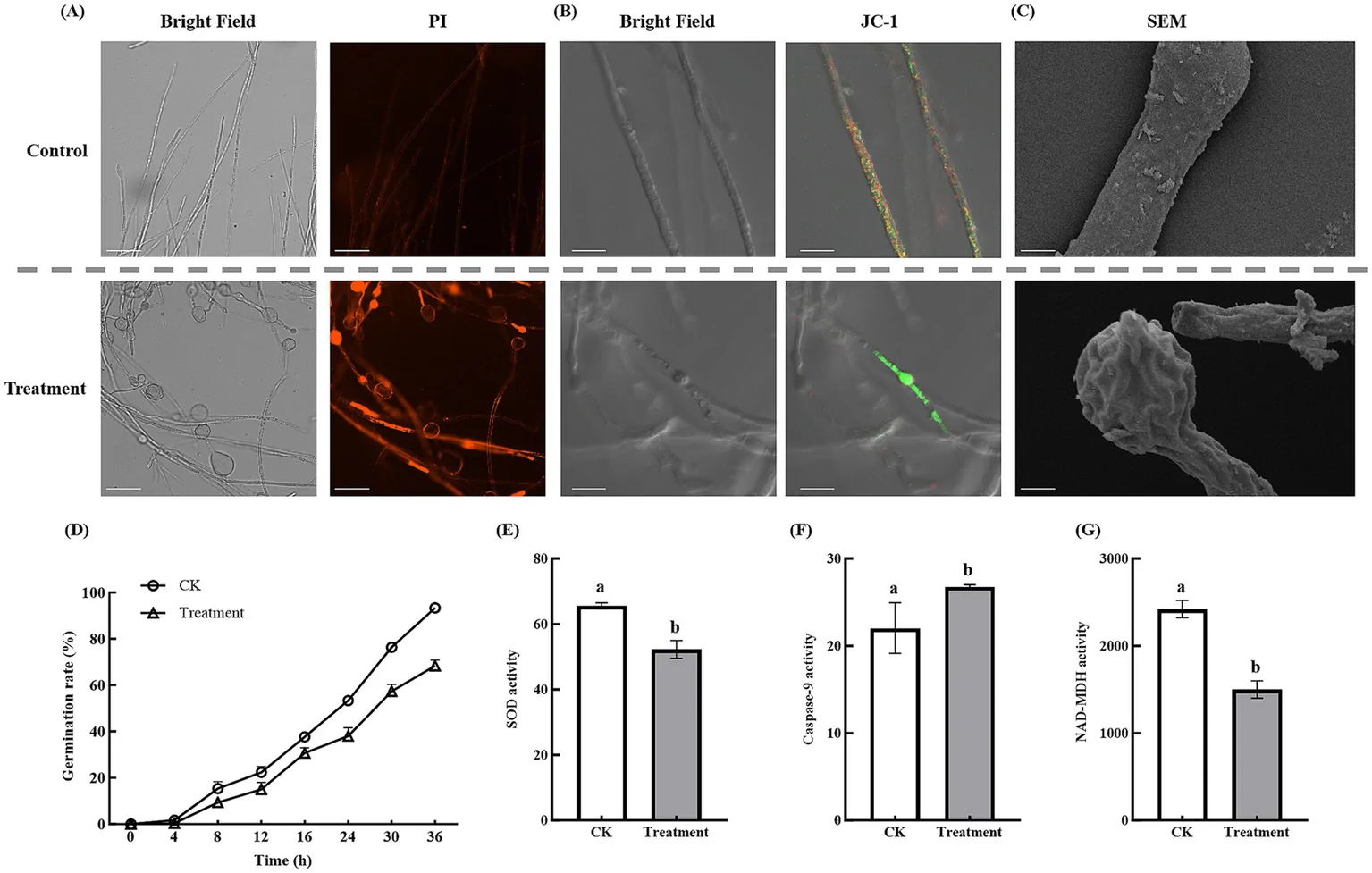
Effects of the crude extract from strain 802 on membrane integrity, mitochondrial membrane potential, hyphal morphology, conidial germination, antioxidant enzyme activities, caspase-9-like activity, and energy metabolism-related enzyme activity of *Aspergillus flavus*. **(A)** Fluorescence microscopy images of PI-stained *Aspergillus flavus* hyphae. **(B)** Confocallaser scanning microscopy images of JC-1-stained *Aspergillus flavus* hyphae. Scale bars, 50 μm. **(C)** Scanning electron microscopy images showing morphological alterations in *Aspergillus flavus* hyphae. Scale bars, 10 μm. **(D)** Effect of the crude extract from strain 802 on the conidial germination of *Aspergillus flavus*. **(E)** Superoxide dismutase (SOD) activity. **(F)** Caspase-9-like activity. **(G)** NAD-dependent malate dehydrogenase (NAD-MDH) activity. Different lowercase letters indicate significant differences between treatments for the same parameter (*p* < 0.05). Control, untreated control; crude extract, treatment with 2% crude extract from strain 802.

SEM images revealed that untreated *A. flavus* hyphae exhibited a regular, smooth, and tubular morphology, whereas hyphae exposed to the crude extract from strain 802 displayed pronounced surface wrinkling, deformation, and localized swelling (Figure 7C). These morphological alterations suggest that exposure to the crude extract disrupted the structural integrity of the hyphal surface and may have affected the cell wall and/or plasma membrane architecture of *A. flavus*. The crude extract from strain 802 also significantly inhibited the germination of *A. flavus* conidia (Figure 7D). After 36 h of incubation, the conidial germination rate in the crude extract-treated group was 25% lower than that in the untreated control. Collectively, these results demonstrate that the crude extract from strain 802 interfered with conidial germination and exerted multiple cellular effects on *A. flavus*, including disruption of plasma membrane integrity, mitochondrial membrane depolarization, and pronounced morphological alterations.

### 3.6 Effect of the crude extract of strain 802 on the antioxidant capacity and energy metabolism of *Aspergillus flavus*

The effects of the crude extract on the antioxidant defense system and cellular energy metabolism of *A. flavus* were evaluated after 4 h of treatment. Compared with the control, SOD activity decreased by approximately 21.30%, indicating that the crude extract disrupted the antioxidant enzyme system of *A. flavus* (Figure 7E). Although caspase-9-like activity exhibited a slight upward trend, the difference was not statistically significant (Figure 7F). In contrast, the activity of NAD-MDH, a key enzyme involved in cellular energy metabolism, decreased by approximately 40% relative to the control (Figure 7G), indicating that the crude extract markedly impaired cellular energy metabolism in *A. flavus*.

### 3.7 Prediction of SMs biosynthesis clusters

To investigate the biosynthetic potential of strain 802, genome mining was performed using antiSMASH to identify BGCs. A total of 13 putative BGCs were predicted in the genome of strain 802 (Table 3). Among these, eight BGCs showed high similarity to previously characterized clusters, whereas the remaining five were predicted as orphan or uncharacterized BGCs. The characterized BGCs included three NRPS-associated clusters corresponding to surfactin, fengycin and bacillibactin, two trans-AT PKS clusters corresponding to macrolactin H and difficidin, one trans-AT PKS/T3PKS/NRPS hybrid cluster corresponding to bacillaene, one PKS-like cluster showing low similarity (7%) to the butirosin A/B cluster, and one cluster corresponding to bacilysin.

**Table 3:** Secondary metabolite clusters predicted in the genome of strain 802.

Cluster name	Start	End	Similar cluster	Similarity (%)	Predicted function	Reference
NRPS	3,038,734	374,141	Surfactin	78	Antiviral effect and antibacterial activity	Vollenbroich et al. (1997)
NRPS, transAT-PK, betalactone	1,852,553	1,990,382	Fengycin	100	Antifungal activity	Hanif et al. (2019)
NRP-metallophore, NRPS, RiPP-like	3,056,411	3,108,204	Bacillibactin	100	Antiviral effect	Xiang et al. (2025)
Terpene	1,0431,161	1,063,856	Unknow	-	Unknown terpene	-
Terpene	2,012,932	2,034,815	Unknow	-	Unknown terpene	-
transAT-PKS	1,378,542	1,466,751	Macrolacti H	100	Antibacterial activity	Chen J. et al. (2019)
transAT-PKS	2,342,594	2,448,766	Difficidin	100	Antibacterial activity	Wu et al. (2015)
transAT-PKS, T3PKS, NRPS	1,688,024	1,798,144	Bacillaene	100	Antibacterial activity	Um et al. (2013)
PKS-like	919,837	961,081	ButirosinA / ButirosinB	7	Bacteriostatic activity	Heifetz et al. (1976) and Heifetz et al. (1972)
Terpene precursor	2,472,076	2,492,966	Unknow	-	Unknown terpene precursor	-
T3PKS	2,142,210	2,183,310	Unknow	-	Unknown T3PKS metabolite	-
Lanthipeptide class II	1,185,771	1,214,660	Unknow	-	Unknown RiPP	-
Other	3,614,574	3,655,992	Bacilysin	100	Antifungal activity	Wu et al. (2014)

### 3.8 Analysis of small SMs

The antibacterial metabolites produced by the antagonistic strain 802 were analyzed using UHPLC-OE480-MS/MS. Based on accurate mass measurements, six putative small-molecule SMs were tentatively identified in the fermentation broth. These compounds included isobutyric acid, lauric acid, MDEP, estradiol valerate, *p*-nitroacetophenone, and ethyl decanoate, with observed *m/z* values of 88.05241, 200.17768, 210.06515, 356.23533, 165.04251, and 200.17775, respectively (Table 4).

**Table 4:** UHPLC-OE480-MS/MS analysis of the components in the crude extract from *Bacillus velezensis* 802.

Compound	Observed *m/z*	Theoretical *m/z*	Molecular formula
Isobutyric acid	88.05241	88.11	C_4_H_8_O_2_
Lauric acid	200.17768	200.32	C_12_ H_24_ O_2_
Methyl diethylphosphonoacetate	210.06515	210.16	C_7_ H_15_ O_5_ P
Estradiol valerate	356.23533	356.5	C_23_ H_32_ O_3_
*p*‑nitroacetophenone	165.04251	165.15	C_8_H_7_NO_3_
Ethyl decanoate	200.17775	200.35	C_12_ H_24_ O_2_

### 3.9 Antifungal activity of small SMs substances against *Aspergillus flavus*

Among the compounds identified in the crude extract of strain 802, the Oxford cup assay was used to evaluate the antifungal activities of six low-molecular-weight compounds, including isobutyric acid, lauric acid, MDEP, estradiol valerate, *p*-nitroacetophenone, and ethyl decanoate, against *A. flavus* (Figure 8). Among the tested compounds, isobutyric acid exhibited the strongest antifungal activity, completely inhibiting the growth of *A. flavus* (100% inhibition), and its inhibitory effect was significantly greater than those of the other compounds (*p* < 0.05). The remaining compounds showed markedly weaker antifungal activities, with inhibition rates of 47.45, 34.51, 29.80, 16.86, and 12.55% for *p*-nitroacetophenone, lauric acid, ethyl decanoate, estradiol valerate, and MDEP, respectively.

**Figure 8:**
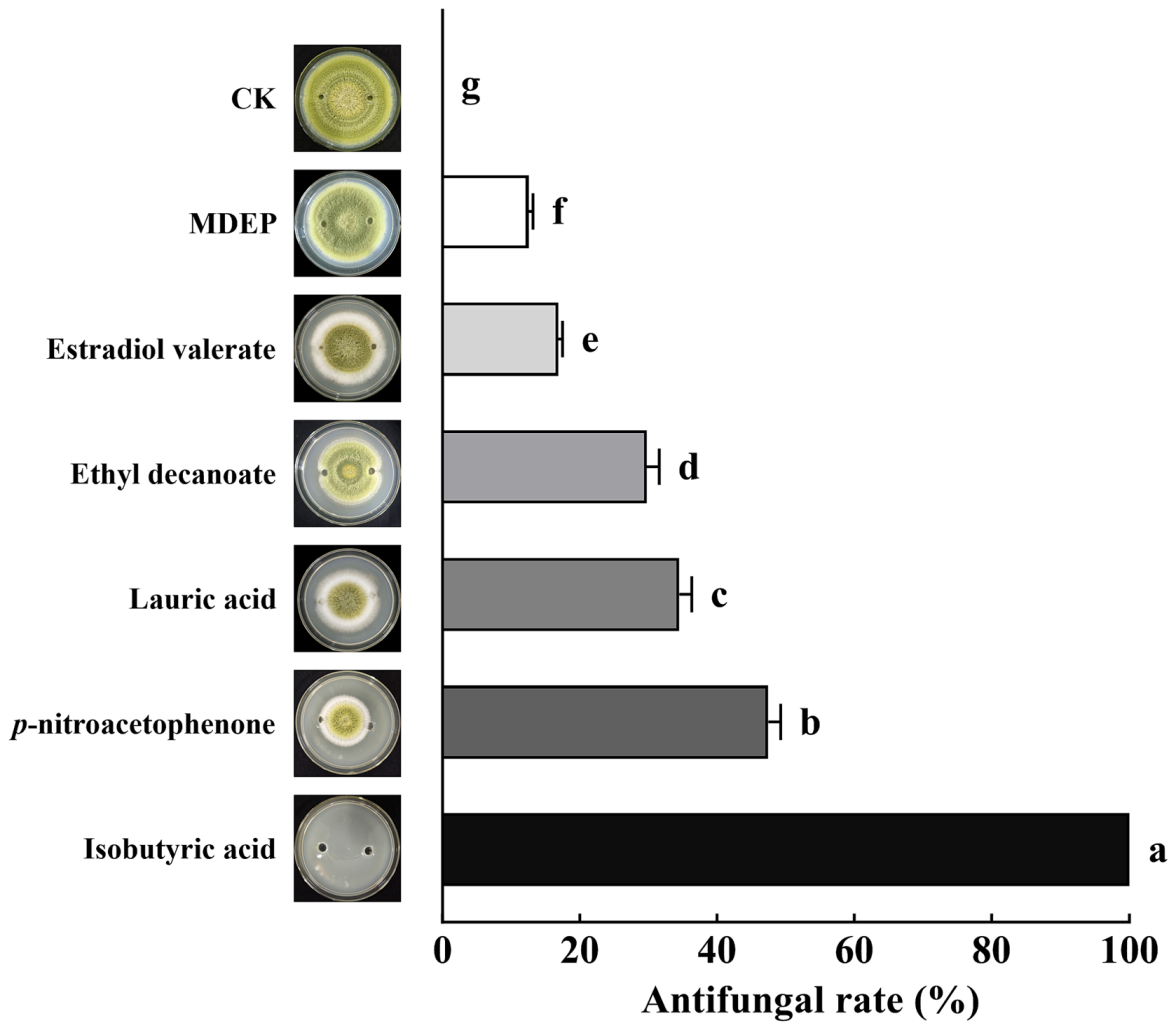
Preliminary screening of the antifungal activities of six secondary metabolites identified in the crude extract of strain 802 against *Aspergillus flavus* using the Oxford cup assay. Isobutyric acid and ethyl decanoate were tested in their undiluted forms, whereas lauric acid, MDEP, estradiol valerate, and *p*-nitroacetophenone were tested at a final concentration of 0.2 mg/mL. Different lowercase letters indicate statistically significant differences among treatments (*p* < 0.05).

## 4 Discussion

*Aspergillus flavus* is a ubiquitous saprophytic fungus that infects cereal grains during both pre-harvest and post-harvest storage, causing grain deterioration and aflatoxin contamination, thereby leading to substantial yield losses and posing a serious threat to food safety (Amaike and Keller, 2011; Mo et al., 2026). Certain *Bacillus* species are capable of producing a diverse array of antimicrobial SMs that suppress plant pathogens, thereby reducing the reliance on synthetic pesticides in sustainable agriculture (Koilybayeva et al., 2023). Consequently, biocontrol agents have emerged as cost-effective, environmentally friendly, and sustainable alternatives for controlling phytopathogenic microorganisms (Moh et al., 2020). Previous studies demonstrated that *B. amyloliquefaciens* ZH409 and *B. velezensis* ZH179 effectively suppressed *A. flavus* through the production of antifungal metabolites (Ma et al., 2025). Similarly, *B. velezensis* E2 exhibited strong inhibitory activity against fungal growth by suppressing spore germination and inducing hyphal abnormalities (Li et al., 2022).

The good antifungal activity of *B. velezensis* 802 against *A. flavus*, together with its effective suppression of fungal proliferation in grain and feed substrates, highlights its potential for controlling *A. flavus* contamination during storage. Consistent with our findings, previous studies have demonstrated that *Bacillus* spp. can effectively suppress *A. flavus* colonization in stored grain matrices and reduce the associated risk of aflatoxin contamination (Centeno et al., 2021; Ma et al., 2025). These findings suggest that the bioactive metabolites produced by strain 802 may provide a promising biological strategy for protecting stored grains and feeds from fungal deterioration and associated mycotoxin risks. Furthermore, rather than acting through a single target, our findings suggest that the antifungal activity of strain 802 involves multiple cellular processes. The significant reduction in mitochondrial membrane potential, together with increased membrane permeability, indicates that the crude extract simultaneously disrupts membrane integrity and mitochondrial function. In addition, inhibition of NAD-MDH, a key enzyme in the tricarboxylic acid cycle, suggests impaired energy metabolism, which is likely to reduce ATP production and restrict fungal growth. Meanwhile, the increased Caspase-9 activity suggests activation of mitochondria-associated apoptosis-related signaling. Therefore, it may be inferred *B. velezensis* 802 exerts its antifungal effect through a multi-target mechanism involving cell wall/membrane disruption, energy metabolism interference, and apoptosis induction.

Genome sequencing combined with antiSMASH analysis revealed that *B. velezensis* 802 harbors multiple BGCs associated with the production of antimicrobial SMs, demonstrating its strong genetic capacity for secondary metabolite biosynthesis. Consistent with the genomic prediction, LC-MS analysis detected several metabolites corresponding to the predicted biosynthetic pathways, providing biochemical evidence that these gene clusters are functionally active under the experimental conditions. Among the identified metabolites, surfactins and fengycins have been widely reported to disrupt fungal membrane integrity by increasing membrane permeability and inducing leakage of intracellular components (Yang et al., 2026), which is consistent with the membrane damage and hyphal deformation observed in the present study. In addition to these lipopeptides, other predicted metabolites, including difficidin, bacillaene, bacillibactin, butirosin and macrolactin H, may also contribute to fungal inhibition through complementary antimicrobial mechanisms. Previous studies have shown that different *Bacillus* strains possess distinct secondary metabolite profiles and therefore exhibit different antifungal activities (Latorre et al., 2016). Therefore, the antifungal activity of strain 802 is more likely attributable to the coordinated action of multiple SMs rather than a single bioactive compound. Similar synergistic antifungal mechanisms involving diverse SMs have also been reported in *B. subtilis* DHA41 (Al-Mutar et al., 2023).

In this study, UHPLC-OE480-MS/MS profiling identified six SMs in *B. velezensis* 802, isobutyric acid, lauric acid, *p*-nitroacetophenone, MDEP, estradiol valerate, and ethyl caprate, revealing a chemically diverse metabolite profile. *Bacillus* species are known to produce a broad spectrum of bioactive metabolites with antifungal properties (Jovana et al., 2023). However, the composition of these metabolites can vary considerably among *Bacillus* strains and under different culture and extraction conditions. Previous study have reported that the extract of *B. mojavensis* contained nine identified compounds accounting for 87.80% of the total extract, with oxygenated monoterpenes predominating, and the major constituents were 3-thujanol and *p*-cymen-8-ol (Camele et al., 2019). This chemical profile differs from those reported for other *Bacillus* strains in which lipopeptides, such as surfactin and iturin, are frequently identified as major bioactive metabolites, suggesting that strain-specific characteristics, culture media, and extraction procedures can substantially influence the metabolite composition of *Bacillus* extracts. Beyond differences in chemical composition, individual SMs may contribute directly to the antifungal activity of *Bacillus*. Volatile metabolites produced by beneficial microorganisms have been shown to suppress *A. flavus* growth and reduce AFB1 contamination in maize and peanuts, highlighting their potential as environmentally friendly antifungal agents (Laurie et al., 2022). Supporting this notion, methyl 2-methylbutyrate has been reported to inhibit *A. flavus* mycelial growth while suppressing aflatoxin biosynthesis (Wei et al., 2024b). Similarly, genome-guided analyses demonstrated that *B. velezensis* LMY3-5 suppresses *Botryosphaeria dothidea* through the production of multiple antimicrobial metabolites, emphasizing the central role of SMs in *Bacillus*-mediated biocontrol (Ren et al., 2025). In the present study, metabolites showed different degrees of inhibition on *A. flavus*. Among them, isobutyric acid had the strongest activity, followed by lauric acid and p-nitroacetophenone. Furthermore, plant-derived antimicrobial compounds have been shown to induce apoptosis-like cell death in *A. flavus*, providing an effective strategy for fungal control (Tian et al., 2018). Collectively, these findings indicate that the bioactive SMs produced by strain 802, particularly antimicrobial lipopeptides and small bioactive compounds, represent promising candidates for developing stable cell-free biocontrol formulations for postharvest disease management and grain storage protection.

## 5 Conclusion

*Bacillus velezensis* 802 exhibits considerable potential as a biological control agent against *A. flavus*. The integrated physiological, genomic, and metabolomic evidence obtained in this study suggests that its antifungal activity is mediated through a multifaceted mode of action rather than a single cellular target. Mechanistically, the crude extract appears to exert its antifungal effects by compromising membrane integrity and mitochondrial function. In particular, the loss of mitochondrial membrane potential, accompanied by increased Caspase-9 activity, suggests a potential link between mitochondrial dysfunction and apoptosis-related processes in *A. flavus.* In addition, the alterations in antioxidant enzyme activities and reduced NAD-MDH activity indicate disturbances in redox homeostasis and cellular energy metabolism. Genome mining identified 13 BGCs associated with antimicrobial secondary metabolite production, while metabolomic analysis detected six bioactive SMs, further demonstrating the substantial biosynthetic potential of strain 802 and providing a molecular and chemical basis for its antifungal capacity. Importantly, the antifungal efficacy of strain 802 in grain and feed matrices highlights its potential for the biological protection of stored commodities. Further integration of transcriptomic and metabolomic analyses may provide deeper insights into the molecular mechanisms underlying its antifungal activity. Future studies should focus on evaluating combinations of the identified bioactive metabolites, elucidating their potential synergistic interactions, and validating their efficacy in suppressing fungal growth and aflatoxin contamination under practical storage conditions.

## Data Availability

The datasets presented in this study can be found in online repositories. The names of the repository/repositories and accession number(s) can be found in the article/supplementary material.

## Footnotes

1
https://tygs.dsmz.de/
